# We now know what fly photoreceptors compute

**DOI:** 10.1186/1471-2202-14-S1-O5

**Published:** 2013-07-08

**Authors:** Uwe Friederich, Stephen A Billings, Mikko Juusola, Daniel Coca

**Affiliations:** 1 Department of Automatic Control and Systems Engineering, University of Sheffield, Sheffield, S1 3JD, UK; 2 Department of Biomedical Science, University of Sheffield, Sheffield, S10 2TN, UK

## 

It has been known for some time that photoreceptors transmit more information when driven by stimuli which have 'naturalistic' statistical properties and that processing of naturalistic visual stimuli involves nonlinear transformations of the input signals. Until now it was not clear which statistical features of the stimuli the neurons are selectively 'tuned' to respond to. Another major difficulty was to elucidate the computational mechanisms which explain differences in coding naturalistic and Gaussian stimuli.

Here we used a functional model of *Drosophila *photoreceptor to fully characterize their underlying computational capabilities. The model was derived based on photoreceptor responses measured *in vivo *using naturalistic input stimuli, which the system experiences in its natural environment. In order to characterize the nonlinear transformations performed by photoreceptors we analytically computed the higher-order or generalized frequency response functions (GFRFs) of the identified nonlinear photoreceptor model. Using this approach, we demonstrate, for the first time that the observed increase in power of photoreceptor response, and shift towards higher frequencies as the mean light intensity increases, is the result of changes in the operating point of the photoreceptor, which produces a change in the shape of the magnitude of the frequency response functions. This suggests that the photoreceptor adaptation mechanisms are not tuned to fully compensate for the drop in intensity in order to achieve, in an efficient way - by exploiting the photoreceptor nonlinearity - a change in the shape of the magnitude transfer functions, which are optimal for the given mean intensity level.

Furthermore, we examined the significance of both local and non-local high-order phase correlations for fly vision. We simulated the photoreceptor model using synthetic stimuli sequences incorporating local phase correlations (edges) and non-local phase correlations (quadratic phase coupling) superimposed with Gaussian white noise. By decomposing voltage output of photoreceptor somata into linear second- and higher-order responses, we explain the nonlinear mechanisms responsible for coding the local and non-local higher-order statistical features in the stimuli as well as improving their signal-to-noise ratio.

To validate the results, we carried out electrophysiological experiments using a specially designed stimuli sequence, which allows extracting the nonlinear component of the photoreceptor response directly from data, without a model.

## Conclusion

The frequency response decomposition employed in this study allowed us to reveal for the first time the quantitative relationship between the higher-order statistical properties of environmental stimuli and processing of these stimuli in fly photoreceptors. In light of the results, we argue that the goal of early sensory coding is to maximize sensitivity to higher-order statistical features of the stimuli that are behaviorally relevant to the animal whilst minimizing sensitivity to non-informative signals, to encode efficiently these features and increase their salience to facilitate further processing. Our framework elegantly explains the differences in coding of naturalistic and white noise signals and how this is achieved efficiently without a change in the response transfer function of the photoreceptor when the mean light intensity is constant. It also explains why and how naturalistic stimuli increase the rate and efficiency of information transmission.

